# Association between internet use and primary headache severity among Hungarian university students: a cross-sectional study

**DOI:** 10.3389/fpubh.2024.1445856

**Published:** 2024-12-16

**Authors:** Ildiko Radvanyi, Antal Tibold, Eva Fejes, Kornel Mak, Szilvia Beke, Gergely Fehér, Rita Nyulas, Valeria Gaal

**Affiliations:** ^1^Centre for Occupational Medicine, Medical School, University of Pécs, Pécs, Hungary; ^2^Hospital of Komló, Komló, Hungary; ^3^Faculty of Health and Social Sciences, Gal Ferenc University, Gyula, Hungary; ^4^Baranya County SZC Zipernowsky Károly Technical College, Pécs, Hungary; ^5^Department of Ophtalmology, Medical School, University of Pécs, Pécs, Hungary

**Keywords:** headache, migraine, tension-type headache, internet use, internet addiction

## Abstract

**Background:**

Recent studies suggest that increased digital technology usage could be a factor in the rising occurrence and severity of headache episodes. The purpose of this cross-sectional study was to determine whether the severity of primary headaches (migraine and tension-type headache) is associated with problematic internet use taking many covariates into account.

**Methods:**

We conducted an online cross-sectional survey using a quantitative, descriptive questionnaire, targeting university students enrolled in correspondence courses, aged 18 to 65. The survey included socio-demographic parameters, risk factors, concomitant diseases, medical history of headaches, and details of online activities. Psychometric measurements contained the Problematic Internet Use Questionnaire, the 9-item short version of the Beck Depression Inventory (BDI-SF), and the Athens Insomnia Questionnaire.

**Results:**

A total of 550 responders (*n* = 480 female; *n* = 70 male) completed the online questionnaire package. Among the participants, 202 individuals (36.7%) reported experiencing headaches, 74 had migraines and 119 had tension-type headache. Internet addiction was detected in 46 (8.4%) participants. Multivariate analysis of variance (MANOVA) showed that significant risk factors of all primary headaches severity included being <30 years (*p* = 0.044, OR = 3.439), not having children (*p* = 0.014, OR = 2.493), being married (*p* = 0.035, OR = 2.528), spending more than 4 h per day on the internet (*p* = 0.021, OR = 1.088), experiencing mood disturbances (*p* = 0.033, OR = 1.345) and the presence of insomnia (*p* = 0.048, OR = 1.667). Furthermore, a slight positive correlation was identified between the amount of time individuals spent on the Internet and the severity of headaches (r = 0.138, r^2^ = 0.019, ß = 1.068, *p* = 0.049). Patients with migraine or tension-type headache showed different predecessors, internet use was only associated with the severity of tension-type headache (night-time internet use, OR = 3.075, *p* = 0.043, and internet addiction, OR = 1.221, *p* = 0.003).

**Conclusion:**

This research marks one of the initial epidemiological investigations in Hungary concentrating on the possible adverse impact of online activities on the severity of headache. Although our study could find slight correlation between the role of online activities and internet addiction on the severity of primary headaches, the topic merits further investigation.

## 1 Background

Primary headache disorders consist of a diverse range of neurological conditions that lead to recurring or persistent head pain without a distinct underlying cause. Headache disorders have become a significant global public health issue. In 2016, it was estimated that nearly three billion individuals experienced either migraine or tension-type headaches: approximately 1.89 billion had tension-type headaches, and 1.04 billion had migraines (1). Although numerous diseases tend to decrease with socioeconomic development, a global analysis indicates an upward trend in the prevalence of primary headaches (1–3). Intense and frequent headaches diminish individuals’ quality of life and contribute to an increase in disability rates over time (4–6). In addition to widely acknowledged lifestyle factors like stress, poor diet and posture, the increased use of digital technology might contribute to the growing frequency and intensity of headache episodes (3, 7).

The internet has become an integral part of our lives, serving as a primary source of information related to work, education, leisure, and communication. While a healthy, controlled use of the web has an undeniably positive impact on our daily lives, excessive, maladaptive use can have a detrimental effect on an individual’s physical health, mental well-being, social relationships, performance at work or school and overall quality of life (8, 9). The problematic usage of internet (PUI) refers to excessive online activities that result in significant functional impairment and/or distress. These activities encompass a range of potentially problematic behaviors related to the internet, such as gaming, gambling, online shopping, cybersex/pornography use, social media engagement, cyberchondria, digital hoarding, cyberstalking, and the excessive use of online streaming, featuring addictive, impulsive, and/or compulsive characteristics (10). The clinical features of activities like gambling or pornography viewing may exhibit phenotypic similarities to behavioral addictions. These behaviors exhibit signs of impaired control, such as unsuccessful attempts to reduce or cease the activity, preoccupation (craving), functional impairment linked to neglect of other life areas, and persistence despite adverse effects (11–14). However, with the exception of gambling disorder, various forms of PUI do not meet the physiological criteria associated with addiction, such as tolerance and withdrawal. Certain manifestations of online shopping or cybersex, may strongly resemble impulse control disorders, and other forms of PUI may exhibit more parallels with obsessive-compulsive disorders (e.g., repeatedly checking emails or social media, digital hoarding) or social anxiety disorder (e.g., excessive reliance on social media as a means of avoiding face-to-face social interactions) (11, 13, 15). However, there is significant overlap in the involvement of addictive, impulsive, and compulsive features across all these types of PUI (10, 11).

The prevalence of Problematic Internet use exhibits considerable variation across studies due to differences in defining the phenomenon, inconsistent criteria, the use of diverse questionnaires, and cultural differences (16). Recent comprehensive analyses indicate that Problematic Internet use could impact around 7% of the population, and research findings consistently report a growing prevalence over successive years (10, 17, 18). According to information from the Hungarian Central Statistical Office, as well as surveys conducted by Gemius and Ipsos, the number of internet users in Hungary has doubled in the past 15 years. Additionally, the time spent online has increased eightfold compared to the preceding period (9, 19, 20). A representative study in Hungary has shown that the prevalence of Problematic Internet use ranges from 1 to 10% in the general population, but possible modifying factors were not mentioned (21). Our recent studies showed that Problematic Internet use could be as high as 20% among secondary school students, and might be approximately 5% in certain adult populations (17, 22–25). The purpose of internet use is also a crucial factor in the development of PUI (10, 26). Engaging in ‘time-wasting’ activities or watching streams can contribute to PUI among middle-aged or older individuals, whereas the prevalence of watching pornography is more common among younger individuals (26).

In addition to the commonly recognized triggers of headache disorders, recent studies have introduced the idea of the spread of digitalization as an emerging factor. Problematic use of internet leads to negative impacts on physical health and somatic symptoms, such as headaches. According to recent literature, a growing number of studies focused on diverse age groups have identified a significant correlation between the duration spent on computer or smartphone screens and the increased frequency and severity of headaches (27–36).

Concerning issues related to headaches associated with PUI, there is limited research, and the findings are inconsistent despite their clinical significance. The connection between PUI and headaches is a topic of debate in the literature. There are studies with conflicting results, the rate of PUI can be significantly lower, similar, or even higher among people suffering from primary headaches compared to controls (37–41) (Table 1). Cerutti et al. in their assessment of Italian children and adolescents, observed no significant correlation between migraines, tension-type headaches and the presence of PUI (37). Similarly, research by Tepecik Böyükbaş et al. involving children found no association between problemtic usage of the internet and headaches (38). In contrast, Corrêa Rangel et al. in a cross-sectional study on evaluating university students identified a significant association between PUI and headache severity (39). Another study by Büsra Demirer et al. highlighted IA as a predictor of headache severity among university students (40). Additionally, a study conducted by Anna Średniawa et al. in Poland found a significant relationship between headache occurrence and the highest level of PUI among students (41).

**Table 1:** Recent studies examining the relationship between internet usage and primary headache disorders.

Study	Type of study	Number and type of participants	Test used to measure Internet PIU	Proportion of PIU	Association between PIU and headaches
Cerutti et al. (37)	Cross-sectional population-based	841 adolescents	Young’s Internet Addiction Test (IAT)	Mobile only abusers: 26.0%; Internet only abusers: 14.9%; Abusers of both media: 19.5%	No significant relationship between headaches and the internet/mobile phone addiction.
Tepecik et al. (38)	Cross-sectional	200 children	Internet Addiction Scale (IAS)	3%	No relationship between IA and headache.
Corrêa et al. (39)	Cross-sectional	420 university students	Brazilian version of Internet Addiction Test (IAT)	20%	Signifcant relationship between IA and headache severity.
Demirer et al. (40)	Cross-sectional	647 university students	Turkish version of Young’s 20-item Internet Addiction Test (IAT)	5.6%	Moderately strong relationship between headache severity and the YIAT score.
Średniawa et al. (41)	Cross-sectional	200 high school graduates	Young’s Internet Addiction Test (IAT)	7%	Significant relationships between the level of Internet addiction and headache.

As there are significant discrepancies in the existing literature (as seen above and in Table 1) we carried out an online survey focusing on the effect of online activities and internet addiction on the severity of primary headache disorders taking many co-variates into account, namely demographical factors, risk factors, history of diseases, depression, sleep disturbance, goals of internet use and problematic usage of the internet.

## 2 Materials and methods

### 2.1 The selection of the study groups

This cross-sectional online survey was carried out between February to May 2023 using a convenience sampling method among students enrolled in correspondence courses of the Gal Ferenc University in Hungary. Participation was anonymous and voluntary. The study protocol and documentation were approved by the Hungarian Mecical Research Council (permission number: BMEÜ /1732–3/2022/EKU).

### 2.2 Data collection tools

Demographic information: included sex, age, marital status, number of children, educational background and secondary employment. Risk factors taken into account were smoking, alcohol intake, history of diabetes, hypertension, cardiovascular disorders, chronic low back pain and depression. Characteristics of internet usage including daily time intervals and the purpose of internet use also were recorded.

Headache Questionnaire: If the participant suffered from headaches in the last 3 months, responses according to lateralization and location of the pain, characteristics and severity of the headache, duration of the attacks, associated symptoms (including nausea, vomiting, photophobia, sensitivity to sound and smell, tearing, red eyes, flushing, nasal congestion, or a runny nose), number of headache days per month, the presence of aura, and factors that either trigger or alleviate the headaches were documented. Headache was diagnosed according to the International Society of Headache (IHS) criteria. Two distinct physicians, a general practitioner (GP) and a neurologist assessed the Headache Questionnaires. Primary headaches were classified into migraine, tension-type headache and unclassified primary headache based on the ICHD-3 diagnostic criteria (42), with the use of the validated Hungarian version of the ID-migraine questionnaire (43). Three levels of headache severity were established. We labeled headaches that occurred infrequently or 1–2 times a month as mild. Moderate headaches were defined as experiencing headaches 1–2 times a week or occasionally having persistent headaches for extended durations followed by headache-free periods. The severe headache category encompassed headaches occurring several times a week or on a daily basis.

As there are no clear diagnostic criteria for internet addiction, it is highly recommended to measure excessive internet use with a continuous questionnaire (7, 22). We chose the Problematic Internet Use Questionnaire (PIU-Q) because its structure tightly adheres to the proposed diagnostic criteria for internet addiction and was created based on the clinometric and psychometric analysis of Young’s internet addiction test, independently validated by several groups and used in our previous published work (7, 23, 25, 44). The questionnaire consists of 18 items, which can be classified into three primary sections: obsession, neglect, and control disorder. Participants rate each item on a 5-point Likert-type scale, ranging from 1 (never) to 5 (always). If the total score exceeds 41 points, it indicates Internet Addiction (Chronbach alpha’s 0.889).

The presence of depression was identified using the 9-item short version of the Beck Depression Inventory (BDI-SF). The questionnaire assesses symptoms such as indecision, social withdrawal, fatigue, sleep disturbance, work incapacity, excessive anxiety about physical symptoms, pessimism, lack of joy, dissatisfaction, and self-blame. Each response is rated on a 4-point Likert scale, ranging from 1 to 4 points (45, 46). Depression can be recognized if the total score exceeds 9 points, and severity can be categorized as mild (10–18 points), moderate (19–25 points), or severe (≥26 points). This questionnaire has been validated in the Hungarian language (46) (Chronbach alpha’s 0.830).

Sleep disturbance was evaluated using the Athens Insomnia Questionnaire, which consists of 8 items addressing both nocturnal symptoms (5 items) and daytime sleepiness (3 items). Responses for each item are rated on a 4-point Likert scale ranging from 0 to 3. A score exceeding 6 points indicated the presence of insomnia (47, 48). This questionnaire has been validated in the Hungarian language as well (48) (Chronbach alpha’s 0.834).

### 2.3 Process and data analysis

Data were evaluated with the use of descriptive statistics, as means ± standard deviation (SD) by using the chi-square test, distribution ratios and correlation ratios. Multivariate analysis of variance (MANOVA) was used to evaluate the outcome changes between groups, with the severity of headaches as independent variables, and demographic data, habits of internet use, PUI, depressive symptoms and sleep disturbance functions as the dependent variables. Mediation analysis was performed for estimation the direct effect of PIU on headache severity and other symptoms. Statistical analysis was carried out with the use of the statistical package of SPSS 11.0 (SPSS, Chicago, IL, USA).

## 3 Results

### 3.1 Sociodemographic characteristics

A total of 550 responders (480 females and 70 males) successfully completed the online survey. Analyzing the age distribution among the study participants, 59% (324) were in the age range of 19 to 40, 40.5% fell between 41 and 60, and the remaining participants were older than 60 years. Among the survey population, 74.9% (412 responders) were either married or in a relationship, and 44.2% (243 responders) did not have children. The proportion of participants with two or more children was 39.3%. Additionally, 84.2% of the study subjects held secondary employment. In terms of substance use, 17.6% of the study population were regular smokers, and 2.4% reported alcohol use (Table 2).

**Table 2:** Sociodemographic data of the study population in aspects of the occurrence and severity of headaches.

	Participants	Occurrence and type of headache			
	Total	Have no headache	Have headache	Migrain	Tension-type headache
	*N* = 550 (%)	*N* = 348 (%)	*N* = 202 (%)	*N* = 74 (%)	*N* = 119 (%)
Gender					
Male	70 (12.7)	55 (15.8)	15 (7.4)	3 (4.1)	11 (9.2)
Female	480 (87.3)	293 (84.2)	187(92.6)	69(95.9)	108(90.8)
Age					
19 years	19 (3.5)	13 (3.8)	6 (3.0)	1 (1.3)	5 (4.2)
20–29 years	172 (31.3)	93 (26.7)	79 (39.1)	27(36.5)	49 (41.2)
31–40 years	133 (24.2)	79 (22.7)	54 (26.7)	20(27.0)	29 (24.4)
41–50 years	169 (30.7)	116 (33.3)	53 (26.2)	23(31.1)	29 (24.4)
51–60 years	54 (9.8)	44 (12.6)	10 (5.0)	3 (4.1)	7 (5.9)
61–65 years	3 (0.5)	3 (0.9)	0	0	0
Marital status					
Single	138 (25.1)	87 (25.0)	51 (25.3)	15(20.3)	34 (28.6)
Relationship	176 (32.0)	97 (27.9)	79 (39.1)	32(43.2)	46 (38.7)
Married	236 (42.9)	164 (47.1)	72 (35.6)	27(36.5)	39 (32.7)
Number of children					
Have no child	243 (44.2)	137 (39.4)	106(52.5)	34(45.9)	67 (56.3)
1 Child	91 (16.5)	65 (18.7)	26 (12.9)	11(14.9)	12 (10.1)
2 Children	131 (23.8)	82 (23.5)	49 (24.2)	19(25.7)	29 (24.4)
More than 3 children	85 (15.5)	64 (18.4)	21 (10.4)	10(13.5)	11 (9.2)
Secondary employment					
No	463 (84.2)	300 (86.2)	163(80.7)	60(81.1)	95 (79.8)
Yes	87 (15.8)	48 (13.8)	39 (19.3)	14(18.9)	24 (20.2)

### 3.2 Type and severity of headache

Within the study population, 36.7% (202/550) reported experiencing headaches, 13.4% (74/550) classified as migraine sufferers and 21.6% (119/550) identified as having tension-type headaches. Among those reporting headaches, 36.6% (74/202) were categorized as migraine sufferers, 58.9% (119/202) were classified as having tension-type headaches, and 4.5% (9/202) were labeled with unclassified primary headaches. The average number of headache days was 13.8. In terms of severity among those individuals suffering from migraine 29.7% (22/74) is classified as mild headache, 29.7% (22/74) as moderate, and 40.6% (30/74) as severe headache. For those with tension-type headaches 47.1% (56/119) is categorized as mild headache, 22.6% (27/119) as moderate headache, and 30.3% (36/119) as severe headache.

### 3.3 Internet use

The average age at which individuals started using digital devices was 17.6 years for the entire study population. A significant portion, 77.6% of responders, reported using the internet for a minimum of 2 h each day. During specific three-hour periods of the day, the preferred time for online activities, based on multiple responses, was predominantly between 6:00 p.m. and 9:00 p.m., as indicated by the majority of participants (57.1%). Regarding the purpose of internet use, based on multiple responses, the highest percentage of individuals (96.5%) used the internet for learning or work, followed by 61.1% who spent most of their time on social networking sites. Additionally, 47.6% of participants used the internet for listening to music, and 45.8% used it for watching movies (Table 3).

**Table 3:** Internet use and problematic internet use in the study population in aspects of the occurrence and severity of headaches (*p < 0.05).

	Participants	Occurrence and type of headache			
	Total	Have no headache	Have headache	Migraine	Tension-type headache
	*N* = 550 (%)	*N* = 348 (%)	*N* = 202 (%)	*N* = 74 (%)	*N* = 119 (%)
Daily internet use (approximately)					
<1 h	23 (4.2)	19 (5.5)	4 (2.0)	0	4 (3.4)
1 h	100 (18.2)	66 (18.9)	34 (16.8)	16 (21.6)	16 (13.4)
2 h	164 (29.8)	110 (31.6)	54 (26.7)	19 (25.7)	33 (27.7)
3 h	115 (20.9)	75 (21.6)	40 (19.8)	14 (18.9)	24 (20.2)
4 h	62 (11.3)	33 (9.5)	29 (14.4)	10 (13.5)	18 (15.1)
5 h	39 (7.1)	21 (6.0)	18 (8.9)	9 (12.2)	8 (6.7)
6 h	16 (2.9)	5 (1.4)	11 (5.5)	4 (5.4)	6 (5.0)
>6 h*	31 (5.6)	19 (5.5)	12 (5.9)	2 (2.7)	10 (8.5) *
Daily time interval of internet use (multiply answer)					
Between 12:00 a.m. and 3:00 a.m.	63 (11.5)	30 (8.6)	33 (16.3)	9 (12.2)	12 (10.1)
Between 3:00 a.m. and 6:00 a.m.	49 (8.9)	30 (8.6)	19 (9.4)	8 (10.8)	11 (9.2)
Between 6:00 a.m. and 9:00 a.m.	99 (18.0)	64 (18.4)	35 (17.3)	11 (14.9)	22 (18.5)
Between 9:00 a.m. and 12:00 a.m.	106 (19.3)	77 (22.1)	29 (14.4)	8 (10.8)	20 (16.8)
Between 12:00 a.m. and 3:00 p.m.	101 (18.4)	63 (18.1)	38 (18.8)	15 (20.3)	22 (18.5)
Between 3:00 p.m. and 6:00 p.m.	160 (29.1)	103 (29.6)	57 (28.2)	20 (27.0)	34 (28.6)
Between 6:00 p.m. and 9:00 p.m.	314 (57.1)	200 (57.5)	114 (56.4)	42 (56.8)	64 (53.8)
Between 9:00 p.m. and 12:00 p.m.*	115 (20.9)	67 (19.3)	48 (23.8)	12 (16.2)	32(26.9)*
Goal of internet use (multiply answer)					
Learning/working	531 (96.5)	339 (97.4)	192 (95.1)	71 (95.9)	112(94.1)
Online gaming	75 (13.6)	52 (14.9)	23 (11.4)	5 (6.8)	18 (15.1)
Chat	316 (57.5)	195 (56.0)	121 (59.9)	43 (58.1)	75 (63.0)
Social media	336 (61.1)	212 (60.9)	124 (61.4)	47 (63.5)	71 (59.7)
Partner search	13 (2.4)	9 (2.6)	4 (2.0)	1 (1.4)	3 (2.5)
Movies	252 (45.8)	147 (42.2)	105 (52.0)	36 (48.6)	63 (52.9)
Music	262 (47.6)	154 (44.3)	108 (53.5)	39 (52.7)	64 (53.8)
Video streaming	64 (11.6)	43 (12.4)	21 (10.4)	7 (9.5)	13 (10.9)
Problematic internet use	46 (8.4)	10 (2.9)	36 (17.8)	9 (12.2)	26 (21.8)

### 3.4 Problematic internet use

Participants were categorized into either Problematic Internet users (experiencing Internet Addiction, IA) or normal internet users (not addicted to the internet) according to the PIU-Q results. As per the definition, 8.4% of the study population falls into the Problematic Internet use (PIU) category. In the headache-free group, 2.9% of responders were detected to have Problematic Internet use (PIU), while in the group experiencing headaches, 17.8% of responders were identified with PIU. This proportion of individuals with Problematic Internet use was 21.8% in the tension-type headache group and 12.2% among migraineurs (Table 3).

### 3.5 Depressive symptoms and insomnia

Depressive symptoms was not identified in 30.3% (167/550) of the participants, whereas 64.3% (354/550) exhibited mild depressive symptoms, 5.0% (28/550) had moderate depressive symptoms, and 0.01% (1/550) experienced severe depressive symptoms based on BDI results. Insomnia was identified in 5.8% (32/550) of the study population (Table 4).

**Table 4:** Depressive symptoms and insomnia in the study population in aspects of the occurrence and type of headaches.

	Participants	Occurrence and type of headache			
	Total	Have no headache	Have headache	Migraine	Tension-type headache
	*N* = 550 (%)	*N* = 348 (%)	*N* = 202 (%)	*N* = 74 (%)	*N* = 119 (%)
Depression					
Have no depressive symtoms	167 (30.4)	127 (36.5)	40 (19.8)	9 (12.2)	29 (24.4)
Mild	354 (64.3)	210 (60.3)	144 (71.3)	55 (74.3)	82 (68.9)
Moderate	28 (5.1)	11 (3.2)	17 (8.4)	10 (13.5)	7 (5.9)
Severe	1 (0.2)	0	1 (0.5)	0	1 (0.8)
Insomnia	32 (5.8)	13 (3.7)	19 (9.4)	8 (10.8)	11 (9.2)

### 3.6 Risk factors of headache severity

Significant risk factors associated with increased headache severity included being under the age of 30 (*p* = 0.044, OR = 3.439), not having children (*p* = 0.014, OR = 2.493), having a married status (*p* = 0.035, OR = 2.528), spending more than 4 h per day on the internet (*p* = 0.021, OR = 1.088), experiencing mood disturbances (*p* = 0.033, OR = 1.345) and the presence of insomnia (*p* = 0.048, OR = 1.667). A slight positive correlation was identified between the amount of time individuals spent on the Internet and the severity of headaches (r = 0.138, r2 = 0.019, ß = 1.068, *p* = 0.049) based on the results of the multivariate analysis (MANOVA).

### 3.7 Risk factors of migraine severity

Participants under the age of 30 showed a higher prevalence of severe migraine compared to those aged 30 and above (46.7% vs. 31.8%, *p* = 0.035, OR = 1.298), and individuals without children exhibited a higher proportion of severe migraine compared to those with children (60.0% vs. 31.8%, *p* = 0.044, OR = 2.806). Singles had a significantly lower occurrence of severe migraine compared to those who were not single (20.6% vs. 28.4%, *p* = 0.041, OR = 2.188; Table 5). Survey results indicated a significant association between migraine severity and moderate depressive symptoms (23.3% vs. 6.7%, *p* = 0.032, OR = 2.600), as well as sleep disturbance (23.3% vs. 0.0%, *p* = 0.020, OR = 5.691) based on the rsults of the multivariate analysis (MANOVA; Table 6).

**Table 5:** Sociodemographic data of the study population in aspects of the severity of migraine attacks (*p < 0.05).

	Have no headache	Severity of migraine			
	Total	Mild	Moderate	Severe	*p*
	*N* = 348 (%)	*N* = 22 (%)	*N* = 22 (%)	*N* = 30 (%)	
Gender					
Male	55 (15.8)	1 (4.5)	0	2 (6.7)	0.097
Female	293 (84.2)	21 (95.5)	22 (100.0)	28 (93.3)	0.342
Age					
19 years	13 (3.8)	0	1 (4.5)	0	0.422
20–29 years*	93 (26.7)	7 (31.8)	6 (27.3)	14 (46.7)	0.035
31–40 years	79 (22.7)	6 (27.3)	7 (31.8)	7 (23.3)	0.162
41–50 years	116 (33.3)	8 (36.4)	7 (31.8)	8 (26.7)	0.972
51–60 years	44 (12.6)	1 (4.5)	1 (4.5)	1 (3.3)	0.930
61–65 years	3 (0.9)	0	0	0	-
Marital status					
Single*	87 (25.0)	3 (13.6)	4 (18.2)	8 (26.7)	0.022
Relationship	97 (27.9)	10 (45.5)	9 (40.9)	13 (43.3)	0.690
Married	164 (47.1)	9 (40.9)	9 (40.9)	9 (30.0)	0.773
Number of children					
Have no child*	137 (39.4)	7 (31.8)	9 (40.9)	18 (60.0)	0.044
1 Child	65 (18.7)	2 (9.1)	6 (27.3)	3 (10.0)	0.357
2 Children	82 (23.5)	6 (27.3)	5 (22.7)	8 (26.7)	0.066
More than 3 children	64 (18.4)	7 (31.8)	2 (9.1)	1 (3.3)	0.116
Secondary employment					
No	300 (86.2)	18 (81.8)	19 (86.4)	23 (76.7)	0.410
Yes	48 (13.8)	4 (18.2)	3 (14.6)	7 (23.3)	0.228

**Table 6:** Depressive symptoms and insomnia in the study population in aspects of the severity of migraine (*p < 0.05).

	Have no headache	Severity of migraine			
	Total	Mild	Moderate	Severe	*p*
	*N* = 348 (%)	*N* = 22 (%)	*N* = 22 (%)	*N* = 30 (%)	
Depressive symptoms					
Have no depressive symptoms	127 (36.5)	4 (18.2)	3 (13.6)	2 (6.7)	0.338
Mild	210 (60.3)	1 (4.5)	17 (77.3)	21 (70.0)	0.188
Moderate*	11 (3.2)	17 (77.3)	2 (9.1)	7 (23.3)	0.032
Severe	0	0	0	0	-
Insomnia*	13 (3.7)	0	1 (4.5)	7 (23.3)	0.020

### 3.8 Mediation analysis (factors of migraine severity)

**Figure 1:**
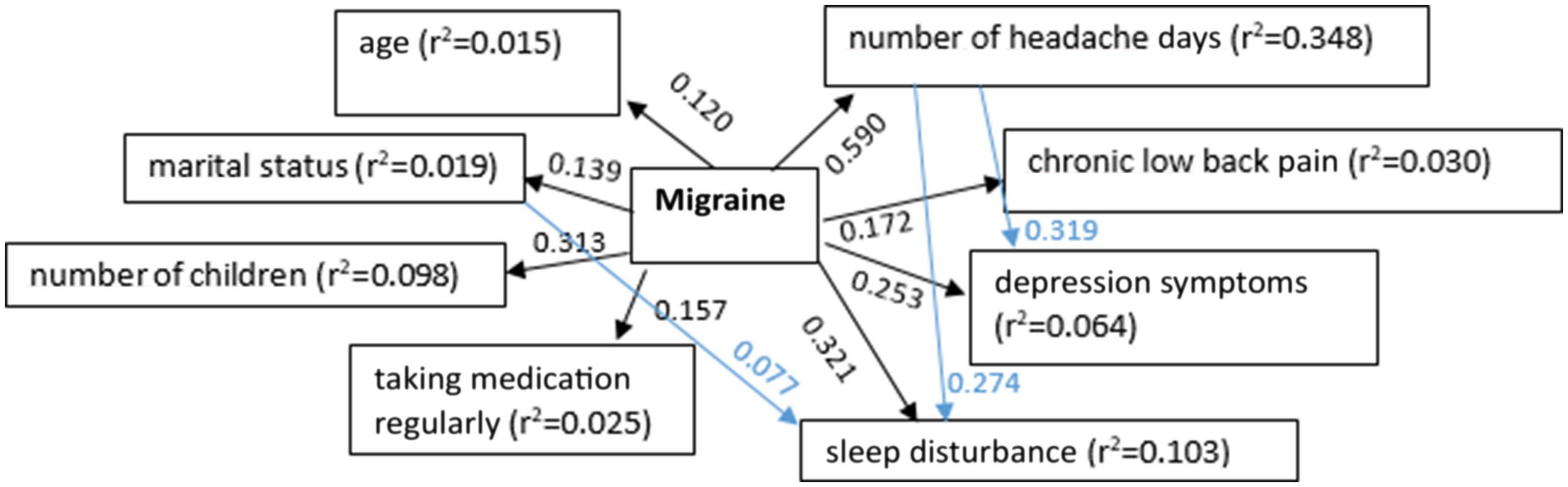
The relationship between migraine and demographic parameters, concomitant diseases, medical history of headaches, and details of online activities (*p* < 0.05).

Mediation analysis model showed that migraine symptoms were influenced by demographic and behavioral factors (e.g., age, marital status, number of children, and regular medication use). Migraine severity was strongly associated with the number of headache days, with additional effects on chronic pain, depression, and sleep quality. There appears to be a feedback loop between migraine and sleep disturbance, where they might reinforce each other over time (Figure 1).

### 3.9 Risk factors of the severity of tension-type headache

Participants under the age of 30 exhibited a higher prevalence of severe tension-type headache compared to those aged 30 and above (50.0% vs. 33.9%, *p* = 0.048, OR = 1.425), and individuals without children had a higher proportion of severe tension-type headache compared to those with children (58.3% vs. 50.0%, *p* = 0.013, OR = 2.528; Table 7). Significant risk factors for tension-type headache were internet use of at least 6 h per day (16.2% vs. 26.9%, *p* = 0.031) and internet use between 21 and 24 h (16.2% vs. 26.9%, *p* = 0.025; Tables 8, 9). Night-time internet use (11.1% vs. 9.0%, OR = 3.075, *p* = 0.043) and internet addiction (33.3% vs. 21.4%, OR = 1.221, *p* = 0.003), as well as mild to moderate depressive symptoms (77.8% vs. 55.4%, *p* = 0.040, 8.3% vs. 3.6%, *p* = 0.024, OR = 1.087) were significantly associated with the severity of tension-type headache according to the survey results based on the rsults of the multivariate analysis (MANOVA).

**Table 7:** Sociodemographic data of the study population in aspects of the severity of tension-type headache (*p < 0.05).

	Have no headache	Severity of tension-type headache			
	Total	Mild	Moderate	Severe	*p*
	*N* = 348 (%)	*N* = 56 (%)	*N* = 27 (%)	*N* = 36 (%)	
Gender					
Male	55 (15.8)	6 (10.7)	2 (7.4)	7 (19.4)	0.417
Female	293 (84.2)	50 (89.3)	25 (92.6)	29 (80.6)	0.169
Age					
19 years	13 (3.8)	0	5 (18.5)	0	0.568
20–29 years*	93 (26.7)	19 (33.9)	12 (44.4)	18 (50.0)	0.048
31–40 years	79 (22.7)	14 (25.0)	4 (14.8)	11 (30.6)	0.551
41–50 years	116 (33.3)	18 (32.1)	5 (18.5)	6 (16.7)	0.465
51–60 years	44 (12.6)	5 (9.0)	1 (3.8)	1 (2.7)	0.224
61–65 years	3 (0.9)	0	0	0	0.505
Marital status					
Single	87 (25.0)	18 (32.1)	10 (37.0)	6 (16.7)	0.492
Relationship	97 (27.9)	20 (35.8)	8 (29.6)	18 (50.0)	0.421
Married	164 (47.1)	18 (32.1)	9 (33.4)	12 (33.3)	0.205
Number of children					
Have no child*	137 (39.4)	28 (50.0)	18 (66.7)	21 (58.3)	0.013
1 Child	65 (18.7)	6 (10.7)	2 (7.4)	4 (11.1)	0.358
2 Children	82 (23.5)	16 (28.6)	5 (18.5)	8 (22.2)	0.058
More than 3 children	64 (18.4)	6 (10.7)	2 (7.4)	3 (8.4)	0.134
Secondary employment					
No	300 (86.2)	48 (85.7)	19 (70.4)	28 (77.8)	0.891
Yes	48 (13.8)	8 (14.3)	8 (29.6)	8 (22.2)	0.656

**Table 8:** Internet use and problematic internet use in the study population in aspects of the severity of tension-type headache (*p < 0.05).

	Have no headache	Severity of tension-type headache			
	Total	Mild	Moderate	Severe	*p*
	*N* = 348 (%)	*N* = 56 (%)	*N* = 27 (%)	*N* = 36 (%)	
Daily internet use (approximately)					
<1 h	19 (5.5)	3 (5.3)	1 (3.8)	0	0.208
1 h	66 (18.9)	11 (19.6)	2 (7.4)	3 (8.3)	0.255
2 h	110 (31.6)	17 (30.3)	6 (22.2)	10 (27.8)	0.721
3 h	75 (21.6)	9 (16.1)	6 (22.2)	9 (25.0)	0.760
4 h	33 (9.5)	7 (12.5)	3 (11.0)	8 (22.2)	0.650
5 h	21 (6.0)	2 (3.6)	6 (22.2)	0	0.716
6 h	5 (1.4)	2 (3.6)	2 (7.4)	2 (5.6)	0.824
>6 h	19 (5.5)	5 (9.0)	1 (3.8)	4 (11.1)	0.469
Daily time interval of internet use (multiply answer)					
Between 12:00 a.m. and 3:00 a.m.	30 (8.6)	6 (10.7)	2 (7.4)	4 (11.1)	0.669
Between 3:00 a.m. and 6:00 a.m.	30 (8.6)	8 (14.3)	3 (11.0)	0	0.942
Between 6:00 a.m. and 9:00 a.m.	64 (18.4)	11 (19.6)	5 (18.5)	20 (55.6)	0.611
Between 9:00 a.m. and 12:00 a.m.	77 (22.1)	8 (14.3)	8 (29.6)	4 (11.1)	0.264
Between 12:00 a.m. and 3:00 p.m.	63 (18.1)	11 (19.6)	3 (11.0)	8 (22.2)	0.873
Between 3:00 p.m. and 6:00 p.m.	103 (29.6)	15 (26.8)	7 (25.9)	12 (33.3)	0.384
Between 6:00 p.m. and 9:00 p.m.	200 (57.5)	28 (50.0)	15 (55.6)	21 (58.3)	0.245
Between 9:00 p.m. and 12:00 p.m.*	67 (19.3)	13 (23.2)	8 (29.6)	11 (30.6)	0.043
Goal of internet use (multiply answer)					
Learning/working	339 (97.4)	56 (100.0)	24 (88.9)	32 (88.9)	0.219
Online gaming	52 (14.9)	6 (10.7)	5 (18.5)	7 (19.4)	0.267
Chat	195 (56.0)	35 (62.5)	20 (74.1)	20 (55.6)	0.497
Social media	212 (60.9)	31 (55.4)	17 (63.0)	23 (63.9)	0.183
Partner search	9 (2.6)	3 (5.4)	0	0	0.337
Movies	147 (42.2)	26 (46.4)	16 (59.3)	21 (58.3)	0.388
Music	154 (44.3)	27 (48.2)	18 (66.7)	19 (52.8)	0.934
Video streaming	43 (12.4)	6 (10.7)	3 (11.0)	4 (11.1)	0.583
Problematic internet use*	10 (2.9)	12 (21.4)	2 (7.4)	12 (33.3)	0.003

**Table 9:** Depressive symptoms and insomnia in the study population in aspects of the severity of tension-type headache (*p < 0.05).

	Have no headache	Severity of tension-type headache			
	Total	Mild	Moderate	Severe	*p*
	*N* = 348 (%)	*N* = 56 (%)	*N* = 27 (%)	*N* = 36 (%)	
Depression					
Have no depression	127 (36,5)	23 (41,0)	2 (7,4)	4 (11,1)	0,203
Mild*	210 (60,3)	31 (55,4)	23 (85,2)	28 (77,8)	0,040
Moderate*	11 (3,2)	2 (3,6)	2 (7,4)	3 (8,3)	0,024
Severe	0	0	0	1 (2,8)	0,330
Insomnia*	13 (3,7)	4 (7,1)	0	7 (19,4)	0,077

### 3.10 Mediation analysis (factors of tension type headache severity)

**Figure 2:**
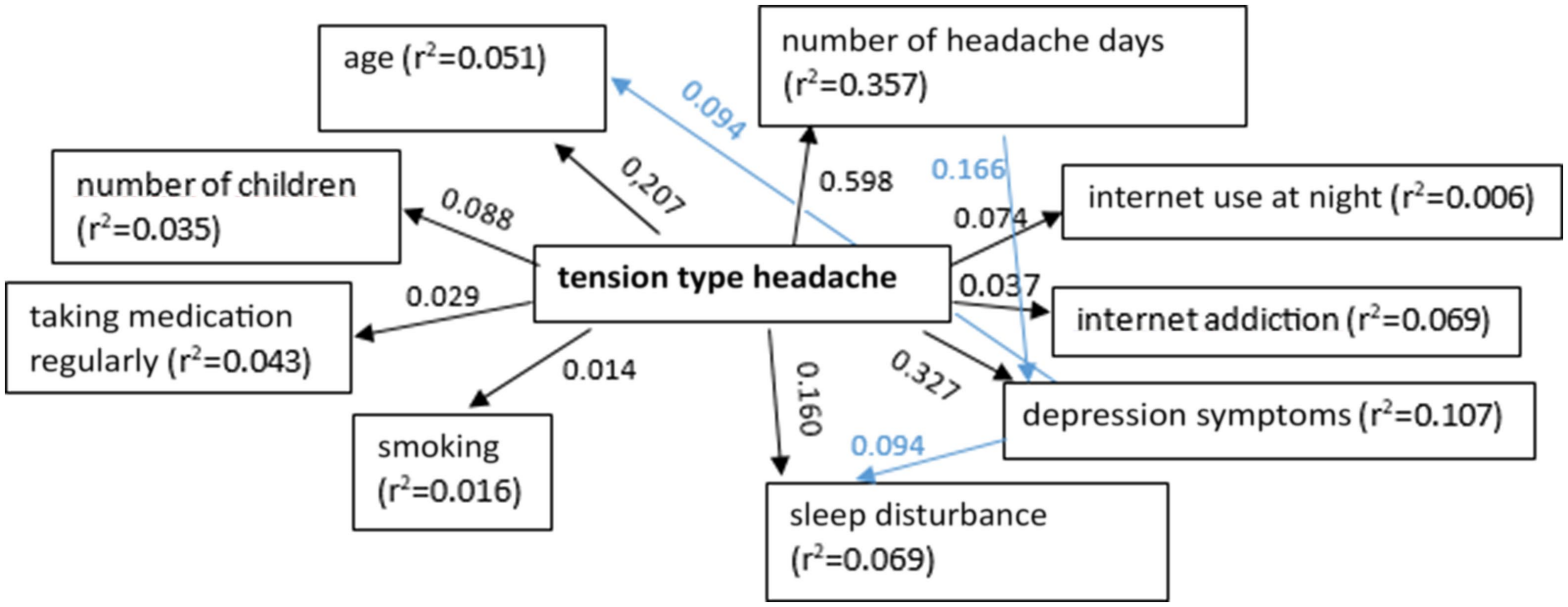
The relationship between tension type headache and demographic parameters, concomitant diseases, medical history of headaches, and details of online activities (*p* < 0.05).

The mediation analysis showed that multiple factors contribute to the development of tension-type headache (TTH), with significant consequences for headache frequency, mental health (depression), sleep quality, and internet usage habits. PUI and depression may partially mediate the relationship between TTH and sleep disturbance. This suggests that effectively managing TTH could benefit from addressing sleep quality, mental health, and internet use habits to break these negative feedback cycles (Figure 2).

## 4 Discussion

Current literature highlights the expanding role of digitalization as an emerging factor linked to adverse effects on physical health, encompassing somatic symptoms such as headaches. In terms of issues related to primary headache disorders associated with Problematic Internet use (PIU), the existing research is limited, and the findings are inconsistent, despite their clinical relevance. The association between Problematic Internet use (PIU) and primary headache disorders is still a subject of discussion in the literature (37–41). The aim of our study was to deepen our understanding of this relationship and assess whether the severity of migraines and tension-type headaches is linked to problematic internet use.

In our study population, the prevalence of primary headaches was found to be 36.7%. In line with established literature, our investigation demonstrated that the prevalence of migraine was 13.4%, and tension-type headache was prevalent in 21.6% of the participants. Epidemiological studies consistently report a higher prevalence of tension-type headache (TTH) compared to migraine (49–51). The global 1-year prevalence for TTH is estimated at 26.8%, while the global 1-year prevalence for migraine is 15.2% in the general population (49, 50).

In our research, we found that being under the age of 30 was identified as a significant risk factor linked to a rise in the severity of headaches. In this instance, we posit the potential influence of stress, which is closely linked to the severity and frequency of headache attacks (52–55). The population of the younger university students differs in various aspects from the general population. This group comprises young adults, a life stage associated with a high prevalence of primary headaches, and is exposed to potential triggers for headache attacks, including stress, the consumption of alcoholic beverages, and sleep deprivation. The presence of these triggers may have contributed to the occurrence of more severe headaches in the 12 months preceding the interview. Moreover, among our student population, there was an increased occurrence of headache comorbidities, including insomnia and depressive symptoms. These factors might have additionally contributed to the heightened frequency of severe headaches, particularly in individuals under the age of 30. Our investigation confirmed that being younger than 30 years old was a significant risk factor associated with increased headache severity in both the migraine and tension-type headache groups in a multivariate analysis.

Another observation from our study reveals that the lack of parenthood emerged as a significant risk factor, correlating with increased severity of headaches. This outcome might be linked to the impact of headaches in women on their decisions regarding pregnancy planning. Results from a multicenter study involving over 600 women with migraines revealed that participants who had more severe and frequent headaches, experienced migraine attacks triggered by menstruation, and had chronic migraines were more likely to decide against pursuing pregnancy. The main reasons for this decision were the concerns about the potential challenges of raising a child while dealing with migraines, the potential negative impact of their migraine medications on their child’s development, and the possibility of their migraine pattern worsening during or shortly after pregnancy; however; we have to face the lack of data in patients with tension-type headache (56). As the vast majority of participants in our study were females, this could be a potential explanation for the association between the lack of children and the severity of headaches. In our study not having children was a significant risk factor linked with heightened headache severity in both the migraine and tension-type headache groups in a multivariate analysis.

We found that marital status is also a significant risk factor correlated with increased severity of headaches. Being married was significantly associated with the severity of migraine, but this connection could not be found within the tension-type headache group. Individuals who were single exhibited a significantly lower prevalence of severe migraines compared to those who were married or in a relationship. We propose that marital stress may play a potential role in explaining this outcome. According to the results of a study, major predictors of response to prophylactic medical therapy in patients with daily migraine headaches involve the patient’s belief that headaches are associated with emotional stress and their unmarried status (57). Unmarried individuals with migraines were more inclined to recognize emotional stress and actively seek psychotherapy.

Daily migraines are likely a response to marital stress. Married individuals who deny the existence of emotional stress in their lives and reject psychotherapy have a lower rate of positive response to prophylactic medical treatment (58). In another study that compared the marital and family adjustment of headache patients and their spouses to couples without chronic pain before pain control treatment, it was observed that headache patients reporting higher marital adjustment were more prone to experiencing persistent pain than those reporting lower marital adjustment. The daily pain reported by headache patients showed a positive correlation with increased family cohesion and adaptability. The severity of pain reported by headache patients positively correlated with greater marital affection. The marital cohesion, affection, family cohesion and adaptability of spouses showed a positive correlation with the heightened severity of patients’ pain (59).

In terms of mood disorders, we observed that the presence of depressive symptoms, as a significant risk factor, was associated with headache severity. We found a higher prevalence of mild and moderate depressive symptoms in the group experiencing headaches in comparison to the headache-free group. In our study the high rate of depressive symptoms among correspondent university students can be attributed to the challenge of juggling work and academic responsibilities, as many of these students are employed full-time while also pursuing their studies. This dual responsibility often results in heightened stress, fatigue, and depressive symptoms. Additionally, students between the ages of 18 and 65 frequently have family and social obligations. Managing family life, childcare, or elder care alongside academic demands can add significant stress. Part-time students also tend to have less frequent interaction with peers and faculty, which may lead to feelings of isolation and a lack of social support, negatively affecting mental health (60–65).

Our findings revealed a significant correlation between the severity of migraines and moderate depressive symptoms as well as between the severity of tension-type headache and mild to moderate depressive symptoms. These results are consistent with the outcomes of studies indicating a higher prevalence of depression in individuals with primary headaches compared to those without headaches (60–62). Moreover, depression has been shown to positively correlate with both the frequency and intensity of headaches (63–65). Specifically, there is a higher prevalence of depression in migraine compared to tension-type headache (62).

In our research, we found that the presence of insomnia was identified as a significant risk factor linked to a rise in the severity of headaches. Sleep disturbance serves as a common trigger for both migraine and tension-type headache (66–68). The frequency of insomnia is elevated in individuals with migraine compared to those without headaches, and both migraine and non-migraine headaches are more prevalent in individuals with insomnia than those without (69). Additionally, the prevalence of insomnia among individuals with tension-type headache is higher than in those without headaches (70).

In addition to the recognized triggers of migraines, recent studies have introduced the idea of digital device use and digital addictions as potential new factors, yielding conflicting results (37–41). In our clinical study, the prevalence of Internet Addiction (IA) was 8.4%, aligning with the findings of a representative study in Hungary, indicating that Problematic Internet use ranges from 1 to 10% in the general population (21). Other recent studies also suggest that Problematic Internet use could be around 5% in specific adult populations (22–25).

We identified a slight positive correlation between the amount of time individuals spent on the Internet and the severity of headaches. Interestingly, these data were driven by those who suffered from tension type headache, multivariate analysis showed that prolonged online being, night-time internet use and internet addiction were significantly associated with the severity of tension-type headache however this association was not corroborated within the migraine group. These findings align with a recent study conducted in Turkey investigating the characteristics of Internet use and Internet addiction among adolescents with headache, which reported that individuals experiencing tension-type headaches spent considerably more time engaging in internet use and playing computer games compared to individuals in the migraine headache group (38). They identified computer use as a more significant headache trigger in individuals with migraines compared to those with tension-type headaches. The differences in our findings regarding the connection between Problematic Internet use and headache severity in the migraine and tension-type headache groups might be clarified by the potentially more prominent triggering role of internet use in the migraine group. Consequently, participants with migraines might use the internet less compared to participants with tension-type headaches to prevent headache attacks.

Mediation analysis showed that several factors contributed to migraine severity, and migraine could have significant consequences for headache frequency, mental health (depression), sleep quality, and chronic pain. Depression partially mediated the effect of migraine on sleep disturbance, and sleep disturbance could exacerbate migraine symptoms in a feedback loop. This suggests that effective migraine management may benefit from addressing sleep quality and mental health support to help break these negative feedback cycles.

This analysis also showed shows that multiple factors contributed to the development of TTH, with significant consequences for headache frequency, mental health (depression), sleep quality, and internet usage habits. Internet addiction and depression may partially mediated the relationship between TTH and sleep disturbance. This suggests that effectively managing TTH could also benefit from addressing sleep quality, mental health as well as internet use habits to break these negative feedback cycles.

The existing literature is slightly controversial on the effect of internet (and problematic internet) use in the severity of headaches. In their very first study Średniawa et al. included 200 high school students in their cross-sectional questionnaire study and found that time spent online and problematic usage of the internet is significantly associated with the prevalence of headaches, however, headache type was not evaluated (42). In their large (including~1,000 participants), cross-sectional study Cerutti et al. concluded that problematic usage of the internet had no impact on both migraine and tension type headache severities (37). They included students aged 10–16 years and the rate of internet addiction was nearly 15%. Interestingly, regular internet users reported higher frequencies of somatic complaints comparing to occasional users (37).

Correa et al. found amongst university students (mean age 21 years), that problematic usage of the internet was associated with anxiety, migraine with aura, and insomnia in a logistic regression model. A significant proportion suffered from PUI, approxymately 20%. Contrary to the above mentioned studies, headache type was determinded by personal interviews using a specific questionnaire, which could lead to more accurate diagnosis. Interestingly, higher frequency and impact of headaches were only associated with PUI in an uni-, but not in a multivariate analysis (39). In another study including univerity students a one-point increase in Young’s Internet Addiction (IAT) test was 1.98 times the risk of experiencing severe headache (40).

In their clinical study Tepercik et al. including children with headaches (mean age 12.6 years) found that internet use was amongst the triggers of headaches, but reccurent headaches were associated with lower rates of internet addiciton, which is slightly contorversial to the above mentioned findings (38).

So the above mentioned studies raised the possible negative effects of internet use and PUI on headache severity, although results are slightly conflicting. Our study is slightly different from its predecessors as our included population was significantly older (more than 60% is above 30 years of age) and we used a more complex methodology (taking more co-variates into account). The used questionnaire was also different as we included the PIU-Q instead of IAT. The rate of PIU was also a bit lower (8.9% instead of 7–20%), which may be due to methodological differences and older age of study participants.

Based on our results significant risk factors of all primary headaches severity included being <30 years, having no children, being married, spending more than 4 h per day on the internet, experiencing mood disturbances and the presence of insomnia, vast majority of these had been identified previously as potential predecessors. Furthermore, a slight positive correlation was identified between the amount of time individuals spent on the Internet and the severity of headaches, which was also not a novelty. Patients with migraine or tension-type headache showed different predecessors, internet use was only associated with the severity of tension-type headache (night-time internet use and internet addiction), which is slightly contrary to the above mentioned studies.

This study represented one of the pioneering epidemiological investigations in Hungary and worldwide focusing on the negative effects of online activities on headache severity among adults. While our findings indicate a modest correlation between problematic internet use and the intensity of primary headaches, further research is essential. Future studies should include longitudinal designs to explore the causal relationship between internet usage patterns and headache severity over time. Comparative studies could also shed light on how various forms of internet engagement (such as social media, gaming, and work-related tasks) impact the severity of different primary headache disorders. Moreover, expanding the research to encompass a broader range of populations beyond university students will help assess the generalizability of these findings.

In conclusion, the extensive utilization of mobile phones and computers has sparked worries regarding the detrimental impact of electromagnetic radiation on human health, creating a noteworthy public health challenge. It is essential to investigate the duration of internet use in patient history interviews. This approach may prove advantageous in identifying the underlying reasons for headaches and implementing strategies for their management. Furthermore, it is advisable to raise awareness among patients and healthcare professionals about potential risk factors to prevent the aggravation of headaches.

This study has several limitations. Data on Problematic Internet use, headache, depression, and insomnia were gathered at a single point in time, preventing the establishment of causal relationships. The study focused on a specific group, namely correspondent university students, which may restrict the generalizability of the findings to other populations. The predominant gender in the sample is female participants, and maintaining gender homogeneity is crucial for unbiased result interpretation. The study was conducted with 480 female and 70 male participants, resulting in a significant gender imbalance, which is considered a limitation of the research. The sample size was not calculated considering the associations between Problematic Internet use and headaches, potentially leading to the study’s inability to detect subtle differences. The extensive use of various questionnaires might have caused participant fatigue. Another limitation of the survey is that 84.2% of the study subjects held secondary employment, but its type was not specified. This additional job type could also potentially impact problematic internet use and headache severity. Despite these limitations, our study has strengths, such as the utilization of validated questionnaires to assess PUI, insomnia, and symptoms of depression. Additionally, the diagnosis of headaches involved two distinct physicians, reducing the likelihood of misclassification.

## Data Availability

The original contributions presented in the study are included in the article/supplementary material, further inquiries can be directed to the corresponding author.
